# Precancerous Cervix in Human Immunodeficiency Virus Infected Women Thirty Years Old and above in Northern Uganda

**DOI:** 10.1155/2016/5473681

**Published:** 2016-07-10

**Authors:** Jonathan Izudi, Norbert Adrawa, Dinah Amongin

**Affiliations:** ^1^Institute of Public Health and Management, International Health Sciences University, P.O. Box 7782, Kampala, Uganda; ^2^Uganda Society for Health Scientists, Department of Anatomy, Makerere University College of Health Sciences, P.O. Box 7072, Kampala, Uganda; ^3^The AIDS Support Organization, TASO Gulu, P.O. Box 347, Gulu, Uganda; ^4^Department of Gynecology and Obstetrics, College of Health Sciences, Makerere University, P.O. Box 7072, Kampala, Uganda

## Abstract

*Background*. Little is known about precancerous cervical lesion (PCCL), the precursor of cervical cancer among Human Immunodeficiency (HIV) infected women in a postconflict setting of Northern Uganda.* Objective*. To establish factors associated with PCCL among HIV infected women above thirty years of age in a postconflict setting of Northern Uganda.* Method*. This retrospective cohort study used electronic data from 995 HIV-positive women that attended cervical cancer screening during June 2014 and December 2015. Data on social, sexual, obstetric, and gynecological factors was analyzed at 95% confidence level. Multivariate analysis determined factors independently associated with positive PCCL. Probability value less than 5% was considered significant.* Results*. Prevalence of PCCL was 3.0% (95% confidence interval (CI): 2.0–4.3). A positive PCCL was significantly associated with absence of sexually transmitted diseases (STDs) during clinic visits (adjusted odds ratio, aOR = 0.24; 95% confidence interval (CI): 0.09–0.64; *P* = 0.004) and first pregnancy before the age of 20 years (aOR = 3.09; 95% CI: 1.21–7.89; *P* = 0.018).* Conclusion*. The prevalence of PCCL was low in the postconflict setting of Northern Uganda. HIV-positive women presenting with STDs and those with first pregnancy before the age of 20 years were at increased risk of PCCL.

## 1. Introduction

Cancer of the cervix is a leading cause of morbidity and mortality among human immunodeficiency virus (HIV) infected women [1]. It usually starts as a small noncancerous growth called precancerous cervical lesion (PCCL) [2, 3]. PCCL takes 10–15 years to become cancerous [2, 3]. In 2012, 14 million new cases of cancer and 8.2 million cancer deaths were recorded in Asia, Africa, and Central and South America. Among the 14 million new cases, cervical cancer accounted for three in every five of the cancer burden [4]. To date, more HIV-positive women suffer from cervical cancers than HIV-negative women [4, 5]. This is because HIV lowers an individual's immunity [6].

Several epidemiological studies linked PCCL to multiple sexual partners, inception of sex at early age, cigarette smoking, and nutritional deficiencies [7]. Meanwhile, other studies implicated Human Papilloma Virus (HPV) infection [8, 9], HIV infection [10], and oral hormonal family planning methods [11]. The progression of PCCL to full-blown cervical cancer is highly preventable when the risk factors are known and screening programs and diagnostic options are available [12].

Current evidence on risk factors for PCCL pertains to the general population of HIV-positive women from stable settings. The application of such findings to make conclusions and interventions among HIV-positive women in unstable settings and among HIV-positive women aged thirty years and above is limiting. In this study, we established factors associated with PCCL among HIV-positive women above thirty years of age attending cervical cancer screening in a postconflict setting of Northern Uganda.

## 2. Methods and Materials

This was a retrospective cohort study that used secondary data collected from HIV-positive women attending cervical cancer screening at the AIDS (Acquired Immune Deficiency Syndrome) Support Organization (TASO), Gulu HIV Clinic [13].

The study cohort comprised of HIV-positive women screened for cervical cancer between June 2014 and December 2015. During the screening, data are collected on sociodemographic, sexual, behavioral, obstetric, and sexually transmitted infections (STIs) using a standardized cervical cancer screening form of Uganda's Ministry of Health. In the HIV clinic, HIV-positive women 15 years and older consent to undergo pelvic examination using a sterile speculum by a trained nurse or midwife. The initial screening technique is Visual Inspection with Acetic Acid (VIA) reported as positive, negative, and uncertain for PCCL. All VIA positive cases undergo colposcopy to confirm PCCL.

**Figure 1:**
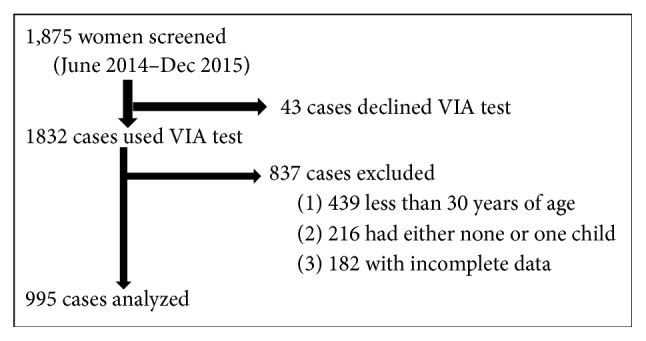
Study profile of HIV infected women screened for cervical cancer (June 2014–December 2015), TASO Gulu.

All cervical screening data are entered in EPIINFO (TM) version 3.4.7. We simply obtained these data, cleaned and exported to Stata version 12 (StataCorp, College Station, TX, USA) for statistical analysis. We analyzed data on 995 HIV-positive women (Figure 1). The outcome variable was positive PCCL test confirmed by colposcopy. The independent variables were sociodemographic, sexual, behavioral, and obstetric, and STI factors. We performed a diagnostic test using Stata version 12 at 95% confidence level to determine sensitivity, specificity, negative predictive value (NPV), and positive predictive value (PPV) of our tests by cross tabulation of VIA test results with colposcopy results.

We stated sensitivity, specificity, PPV, and NPV with corresponding 95% confidence intervals (CI).

We summarized numerical data in frequencies, percentages, means with standard deviations (SD), and medians with interquartile ranges (IQR). The prevalence of PCCL was determined by dividing the number of positive colposcopy tests by the entire sample size, expressed in percentage.

The association between the outcome variable and categorical independent variables was assessed using the Chi-squared test for larger cell counts (above or equal to five) and Fisher's exact test for smaller cell counts (less than five). Student's *t*-test was used for testing association between the outcome variable and numerical independent variable. Chi-squared, Fisher's exact, and Student's *t*-test probability values (*P* values) less than 20% were considered statistically significant for univariate analysis. Meanwhile, *P* values less than 5% at univariable analysis together with clinically relevant variables were considered statistically significant for multivariable analysis using binary logistic regression. Results of binary logistic regression were stated in odds ratios with 95% CI and *P* values.

Ethical approval to use the cervical cancer data was sought from the Ethics and Research Committee of TASO Gulu. All clients had written informed consent at enrollment into HIV chronic care. All TASO clients are identified by unique registration numbers to ensure anonymity, secrecy, privacy, and confidentiality of information.

## 3. Results

### 3.1. Sociodemographic Characteristics of Respondents

The mean age of the 995 women is 40.0 years (SD = 8.2). Among 741 women aged 30–44 years, 21 (2.8%) had PCCL and 720 (97.2%) had no PCCL. Among 222 women in the age group of 45–59 years, 215 (96.9%) had no PCCL and 7 (3.2%) had PCCL. For the age group of 60–70 years, out of 32 women, 30 (93.8%) had no PCCL and 2 (6.3%) had PCCL.

Of 25 women that had their first sexual intercourse before start of menstruation, none (0.0%) had PCCL. 11 (1.8%) out of 622 women that had their first sexual intercourse after start of menstruation had PCCL and 611 (98.2%) had no PCCL. Of 881 women that had less than or equal to five lifetime sexual partners, 29 (3.3%) had PCCL while 852 (96.7%) had no PCCL. On the other hand, of 46 women that had more than five lifetime sexual partners, 1 (2.2%) had PCCL while 45 (97.8%) had no PCCL.

859 women had less than or equal to five sexual partners preceding their clinic visit, of which 26 (3.0%) had PCCL and 833 (97.0%) had no PCCL. On the contrary, among eight women that had more than five sexual partners preceding the clinic visit, 1 (12.5%) had PCCL while 7 (87.5%) had no PCCL. Of 315 women that experienced first menstrual cycle before or at the age of 14 years, 7 (2.2%) had PCCL and 308 (97.8%) had no PCCL. Equally, among 372 women that experienced their first menstrual cycle after the age of 14 years, 7 (1.9%) had PCCL while 365 (%98.1) had no PCCL. Of 46 women that had menopause before the age of 55 years, only 1 (2.2%) had PCCL and 45 (97.8%) had no PCCL.

Three women (100.0%) had menopause after the age of 55 years and all had no PCCL. 20 (3.7%) out of 545 women that had ever got pregnant for less or exactly five times had PCCL. In contrast, 10 (2.3%) out of 427 women that had ever got pregnant for more than five times had PCCL and the 417 (97.7%) had no PCCL. 26 (3.5%) out of 739 women that had less than or equal to five live births had PCCL and 713 (96.5%) had no PCCL. Four (1.8%) out of 224 women that had more than five live births had PCCL while 220 (98.2%) had no PCCL (Table 1).

### 3.2. Prevalence of PCCL

63 (6.3%) out of 995 women tested positive for PCCL with VIA. By colposcopy, 28 out of the 63 cases (3.0%, 95% CI: 2.0–4.3) were confirmed as PCCL-positive cases. The sensitivity of the test was 93.3% (95% CI: 77.9–99.2), specificity was 96.4% (95% CI: 95.0–97.5), PPV was 44.3% (95% CI: 36.2–52.8), and NPV was 99.8% (99.2–99.9). The prevalence of PCCL was therefore 3.0% (95% CI: 2.0–4.3) (Table 2).

### 3.3. Factors Associated with PCCL among HIV-Positive Women Aged 30 Years and Older

Our study participants presented to the HIV clinic cervical cancer screening with sexually transmitted disease (STD) symptoms and concerns (Supplementary Material S1 in Supplementary Material available online at http://dx.doi.org/10.1155/2016/5473681). 111 (11.2%) reported vaginal itching, of which 5 (4.5%) had PCCL and 106 (95.5%) had no PCCL. 854 (85.2%) had no vaginal itching, of which 25 (2.9%) had PCCL and 829 (97.1%) had no PCCL. 48 (5.2%) reported pain during sexual intercourse or during urination, of which 1 (2.1%) had PCCL and 47 (97.9%) had no PCCL.

Of 879 (94.8%) that reported no pain during sex or urination, 28 (3.2%) had PCCL and 848 (96.8%) had no PCCL.

In bivariate analysis, presenting to the HIV clinic with an STD (Chi-squared test = 5.29; degree of freedom (df) = 1; *P* = 0.021), first pregnancy before or after the age of 20 years (Chi-squared test = 6.10; df = 1; *P* = 0.013), and the mean parity (Student's *t*-test = 2.0; df = 970; *P* = 0.046) were significantly associated with PCCL. The average number of sexual partners in the previous month (Fisher's exact test, *P* = 0.07) and the number of lifetime sexual partners (Student's *t*-test = −0.35; df = 925; *P* = 0.728) were considered as clinically relevant variables for PCCL.

In univariable analysis, three factors (presence of an STD, first pregnancy before the age of 20 years, and parity) were statistically significantly associated with PCCL. Women that had no STD during HIV clinic visits were 65% less likely to have PCCL compared to those that had an STD (unadjusted odds ratio (uOR) = 0.35; 95% CI: 0.14–0.89; *P* = 0.027). On the other hand, women that had their first pregnancy before the age of 20 years were over two times more likely to have PCCL compared to women that had had their first pregnancy after the age of 20 years (uOR = 2.88; 95% CI: 1.20–6.94; *P* = 0.018). Also, for every 1-unit increase in parity, the odds of PCCL reduced by 17% (uOR = 0.83; 95% CI: 0.69–1.00; *P* = 0.018). In addition, for every 1-unit increase in the number of lifetime sexual partners, the odds of PCCL increased too (uOR = 1.03; 95% CI: 0.86–1.24; *P* = 0.728).

After adjustment for significant variables at univariate analysis (presence of STDs, first pregnancy before the age of 20 years, and parity) and clinically relevant variables (number of sexual partners in a single month preceding HIV clinic visit and number of lifetime sexual partners), multivariate analysis (Table 2) indicated that women that had no STDs at the time of HIV clinic visit had lower adjusted odds of PCCL compared to women that had STDs (aOR = 0.24; 95% CI: 0.09–0.64; *P* = 0.004). Also, women that had their first pregnancy at or before the age of 20 years had lower adjusted odds of PCCL compared to those that had had their first pregnancy after the age of 20 years (aOR = 3.09; 95% CI: 1.21–7.89; *P* = 0.018). Increase in parity (aOR = 0.88; 95% CI: 0.72–1.07; *P* = 0.198), the number of sexual partners in the preceding month of clinic visit (aOR = 2.75; 95% CI: 0.59–12.71; *P* = 0.196), and the number of lifetime sexual partners (aOR = 1.14; 95% CI: 0.93–1.39; *P* = 0.211) were not associated with PCCL (Table 3).

## 4. Discussion

This study determined factors associated with PCCL among HIV-positive women attending chronic care at TASO Gulu. The prevalence of PCCL was 3.0% (95% CI: 2.0–4.2). Compared to previous studies [5, 10, 14], this was surprisingly low. Earlier studies among 715 HIV-positive women aged 18–69 years found 26.7% prevalence of PCCL in Kenya [10], 22.1% among 448 HIV-positive women in Southern Ethiopia [15], and 13.6% among 301 HIV-positive women in Uganda [14] and 6% in Nigeria [5]. The studies in Kenya, Southern Ethiopia, and Uganda all have smaller sample size compared to the present study. The smaller sample sizes hence account for the higher prevalence of PCCL compared to the present study. The study in Kenya was among HIV-positive women aged 18–69 years unlike the present study that involved HIV-positive women aged 30–70 years. Studies in Southern Ethiopia and Mulago Hospital, Uganda, confirmed reduced prevalence of PCCL among HIV-positive women on ART [14, 15]. The low prevalence of PCCL in this study could be because some of the study participants were on ART. Secondly, this was routinely collected data for monitoring health service delivery and may thus lack the rigor required for research.

In this study, the prevalence of PCCL was 2.8% among HIV-positive women aged 30–44 years, 3.2% among 45–59-year-olds, and 6.3% among 60–70-year-olds. The prevalence of PCCL increased with increasing age. Old age is known to increase the risk of PCCL according to previous literature [16, 17]. Cervical cancer screening interventions should therefore target all HIV-positive women throughout their reproductive years.

Our study indicated that women that had their first pregnancy before the age of 20 years were at increased risk of PCCL. This implies that early pregnancy and early sex is a risk factor for PCCL. Our result confirms the results of previous study in Spain and Columbia that found over fourfold higher risk of cervical cancer among women with history of early (before the age of 16 years) sexual intercourse [18]. Sexual intercourse at an early age increases the risk of multiple lifetime sexual partners and the acquisition of numerous STIs including cervical cancer causing HPV. In addition, the combination of cervical changes during puberty and early sex increases damage to cervical lining [19]. This result confirms the results of past studies [2, 3, 7, 20]. Current prevention interventions should target prevention of HIV-positive adolescent girls from indulging into premarital sex to reduce future risk of cervical cancer.

Women that presented with sexually transmitted diseases (STDs) syndromes such as lower abdominal pain and abnormal and foul smelling vaginal discharges at the HIV clinic were at increased risk of having PCCL. Abnormal vaginal discharge is not a known symptom or concern suggestive of PCCL other than occasional abnormal bleeding but is an important reason for seeking medical care [2]. Abnormal vaginal discharge was found associated with squamous cell carcinoma in Nigeria [21]. It is important to note that STDs precede unprotected sexual intercourse and unprotected sexual intercourse is a risk factor for acquisition of PCCL causing HPV. In Ethiopia, women that came for medical care with an STD had PCCL [15]. In Nigeria, HIV-positive women with vaginal abnormalities had higher risk of PCCL [5].

Literature indicates that as the number of children born to a women increases, the chances of developing cervical cancer increase too [17]. In this study, parity (number of children ever born), increase in the number of lifetime sexual partners, and increase in the number of sexual partners before HIV clinic visit for cervical cancer screening were not associated with PCCL. Our result is contrary to existing literature [17, 22]. It appears that the prevalence of PCCL in HIV-positive women is driven by immunosuppression other than sexual related factors [6].

Our study is the first in Uganda to determine the magnitude of PCCL among HIV-positive women aged 30 years and older in a postconflict setting of Northern Uganda. It highlighted low prevalence of PCCL. The main factors associated with this prevalence were having STDs and first pregnancy before the age of 20 years. We recommend that current HIV prevention interventions should focus on reducing early sexual practices among HIV-positive adolescents to protect them from PCCL. In clinical practice, healthcare providers (physicians, nurses, and medical doctors) should routinely evaluate women presenting with STDs for PCCL or cervical cancer.

Although the present study is limited by lack of data on ART and socioeconomic variables, it has set the benchmark for future research on PCCL among HIV-positive older women (aged thirty years and older). The interpretations of this study should hence recognize these limitations.

## Supplementary Material

Symptoms and concerns reported by study participants during cervical cancer screening.

## Figures and Tables

**Table 1:** Sociodemographic characteristics of respondents by PCCL.

	HIV-positive women with PCCL		
	No	Yes	
Characteristics	Number (%)	Number (%)	Total
Age (years)			
30–44	720 (97.2)	21 (2.8)	741
45–59	215 (96.9)	7 (3.2)	222
60–70	30 (93.8)	2 (6.3)	32
Mean ± SD	40.0 ± 8.2	40.9 ± 7.9	995
Sexual debut			
Before menses	25 (100.0)	0 (0.0)	25
After menses	611 (98.2)	11 (1.8)	622
Number of life partners			
Less than or equal to 5	852 (96.7)	29 (3.3)	881
Greater than 5	45 (97.8)	1 (2.2)	46
Mean ± SD	3 ± 2	3 ± 3	927
Number of sexual partners in thirty days			
Less than or equal to 5	833 (97.0)	26 (3.0)	859
Greater than 5	7 (87.5)	1 (12.5)	8
Mean ± SD	1 ± 1	2 ± 3	867
Age at menarche (years)			
Less than or equal to 14	308 (97.8)	7 (2.2)	315
Greater than 14	365 (98.1)	7 (1.9)	372
Mean ± SD	14.8 ± 1.6	14.8 ± 1.8	687
Age at menopause (years)			
Less than 55	45 (97.8)	1 (2.2)	46
Equal to or greater than 55	3 (100.0)	0 (0.0)	3
Number of pregnancies ever had			
Less than or equal to 5	525 (96.3)	20 (3.7)	545
Greater than 5	417 (97.7)	10 (2.3)	427
Mean ± SD	5 ± 2	4 ± 1	972
Number of live births			
Less than or equal to 5	713 (96.5)	26 (3.5)	739
Greater than 5	220 (98.2)	4 (1.8)	224
Mean ± SD	4 ± 2	3 ± 1	963
Ever smoked cigarette			
Yes	1 (100.0)	0 (0.0)	1
No	943 (97.0)	29 (3.0)	946

**Table 2:** VIA screening results versus colposcopy examination results for PCCL.

Results		VIA screening test		Total
		Positive for PCCL	Negative for PCCL	
Colposcopy confirmation	Positive for PCCL	28	2	*30*
	Negative for PCCL	35	930	*965*

	*Total *	*63*	*932*	*995*

**Table 3:** Sexual and obstetric factors associated with PCCL in multiparous HIV infected women above thirty years old.

Characteristics	HIV-positive women with PCCL?		Univariable analysis	*P* value	Multivariable analysis	*P* value
	No (*n* = 925)	Yes (*n* = 30)	^a^uOR (95% CI)		^b^aOR (95% CI)	
Presence of STDs						
Yes	75 (92.4)	6 (7.4)	1		1	
No	850 (97.2)	24 (2.8)	0.35 (0.14–0.89)	0.027	0.24 (0.09–0.64)	0.004
Had the first pregnancy before the age of 20 years						
No	92 (92.9)	7 (7.1)	1		1	
Yes	834 (97.4)	22 (2.5)	2.88 (1.20–6.94)	0.018	3.09 (1.21–7.89)	0.018
Parity						
1-unit increase in parity (mean ± SD)	5.38 ± 0.08	4.53 ± 0.35	0.83 (0.69–1.00)	0.047	0.88 (0.72–1.07)	0.198
Number of sexual partners in a single preceding month						
Less than or equal to one	811 (97.1)	24 (2.9)	1		1	
More than one	29 (90.6)	3 (9.4)	3.50 (0.20–12.28)	0.051	2.75 (0.59–12.71)	0.196
Number of sexual partners in life						
1-unit increase in the number of sexual partners (mean ± SD)	2.92 ± 0.06	3.03 ± 0.62	1.03 (0.86–1.24)	0.728	1.14 (0.93–1.39)	0.211

*Note*. Percentages were calculated for row totals as *n*/*N*.

CI, confidential interval; uOR, unadjusted odds ratios, aOR, adjusted odds ratios; PCCL, precancerous cervical lesions.

^a^Unadjusted odds ratios for variables with *P* value <0.2 at bivariate level and clinically relevant variables (number of sexual partners in the preceding month and number of lifetime sexual partners).

^b^Multivariable (adjusted) analysis for presence of STDs, first pregnancy before the age of 20 years, parity, number of sexual partners preceding a month, and number of lifetime sexual partners.
