# Supplementary material for: Precancerous Cervix in Human Immunodeficiency Virus Infected Women Thirty Years Old and above in Northern Uganda
Source: J Oncol. 2016 Jul 10;2016:5473681. doi: 10.1155/2016/5473681 (PMC4958448; doi:10.1155/2016/5473681)
Supplement: Supplementary file 1 — Symptoms and concerns reported by study participants during cervical cancer screening. [file 5473681.f1.pdf]

Supplementary Material S1: Symptoms and concerns reported during cervical cancer screening

	HIV-positive women with PCCL			
**Characteristic**	**No**<br>**No. (%)**	**Yes**<br>**No. (%)**	**Total (%)**	**P-value**
**Menstrual flow**				0.25
Heavy	52 (96.3)	2 (3.7)	54	
Moderate	742 (96.6)	26 (3.4)	768	
scanty	21 (91.3)	2 (8.7)	23	
**Vaginal/valval itching**				0.37
Yes	106 (95.5)	5 (4.5)	111	
No	829 (97.1)	25 (2.9)	854	
**Fever**				0.23
Yes	27 (93.1)	2 (6.9)	29	
No	888 (96.9)	28 (3.1)	916	
**Pain during intercourse or urination**				1.00
Yes	47 (97.9)	1 (2.1)	48	
No	848 (96.8)	28 (3.2)	876	
**Bleeding during sex**				1.00
Yes	29 (100.0)	0 (0.0)	29	
No	914 (96.8)	30 (3.2)	949	
